# Temporomandibular joint (TMJ) disorders as first clinical manifestations in external auditory canal cholesteatoma. A case report

**DOI:** 10.1016/j.amsu.2022.103287

**Published:** 2022-01-25

**Authors:** Fatemeh Salimi, Dema Motter, Zahra Salimi

**Affiliations:** a University of Oxford, United Kingdom; b Grange University Hospital, United Kingdom; c University Hospital of Wales, United Kingdom

**Keywords:** Cholesteatoma of the external ear canal, Erosion of the external auditory canal, Otorrhea

## Abstract

**Introduction and importance:**

Cholesteatoma is a common occurrence in the middle ear, whereas cholesteatoma of the external auditory canal (EAC) is a rare condition. We report an unusual presentation of the cholesteatoma in the EAC.

**Case presentation:**

We report a case of a 67-year-old male presented to the ENT casualty with a longstanding history of left sided squeaky type sound, aggravated whenever he talks or eats. He subsequently had a Computed Tomography (CT) scan of the left petrous bone which identified a left-sided EAC cholesteatoma. Clinical symptoms of EAC cholesteatoma are non-specific, and hence we recommend considering cholesteatoma when patients present with abnormal EAC symptoms and intact tympanic membrane.

**Clinical discussion:**

His cranial nerves examination was normal, and the tympanic membrane was intact. His blood count and infective marker were normal. The CT scan of the brain showed a lesion in the left external auditory canal close to the tympanic membrane. The lesion was in contact with the anterior inferior canal wall which had features suggesting bony erosion. Gas bubble seen in the posterior part of the TMJ was in relation to bony erosion of the EAC.

**Conclusion:**

The cholesteatoma of the EAC is very rare. CT scan can provide detailed information about the extent of external ear canal cholesteatoma, which can be used to identify complications of the disease, in addition to differentiating the external ear canal from the middle ear cholesteatoma. Early recognition of cholesteatoma and prompt treatment is essential to prevent catastrophic complications.

## Highlights

• A cholesteatoma originating in the external auditory canal is a rare finding.
• Cholesteatoma can present with atypic symptoms such as temporomandibular joint disorders.

## 1 Introduction

A cholesteatoma consists of a mass of stratified keratinising squamous epithelium in the ear [1]. The aetiology of cholesteatoma is often debated, but acquired cholesteatoma is considered as a lesion that arises from the lateral epithelium of the tympanic membrane, which then grows as a self-perpetuating mass into the middle ear [2]. This lesion can activate the local osteoclasts and lead to serious local tissue destruction. The most common symptoms of the cholesteatoma are headache and hearing loss and less commonly causes otorrhea, eardrum perforation, disequilibrium, facial nerve dysfunction, tinnitus, and epilepsy spells [2,3]. We are presenting a case of cholesteatoma with erosion of the external canal wall leading to gas bubble formation in the posterior of the temporomandibular joint (TMJ) disorders.

This case report has been reported in line with the SCARE criteria [4].

## 2 Case presentation

A 67-year-old man presented with a left sided audible high-pitched sound on TMJ movements. At the presentation he did not have any TMJ dysfunction, trismus, headache or preauricular swelling. He did not complain of hearing loss, or any symptoms of facial palsy. He complained of longstanding unbearable headache, otalgia, and localised TMJ pain which lasted 6 weeks prior to his initial presentation. He did not have a significant past medical history and was not taking any regular medication. There was no significant family history.

**Fig. 1:**
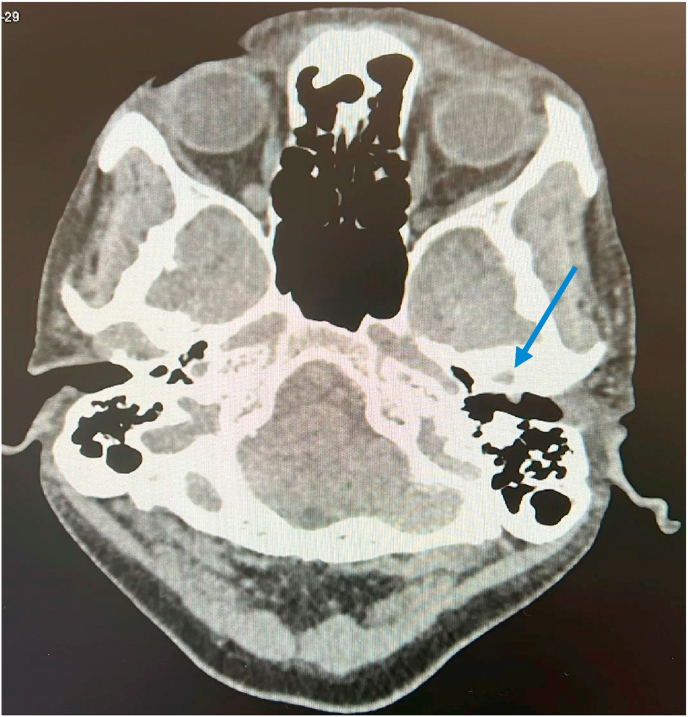
Computerised tomography of the petrous bone. Blue arrow shows 5mm structure in the left external auditory canal near the tympanic membrane. It is in contact with the anterior inferior canal wall which looked eroded. (For interpretation of the references to colour in this figure legend, the reader is referred to the Web version of this article.)

**Fig. 2:**
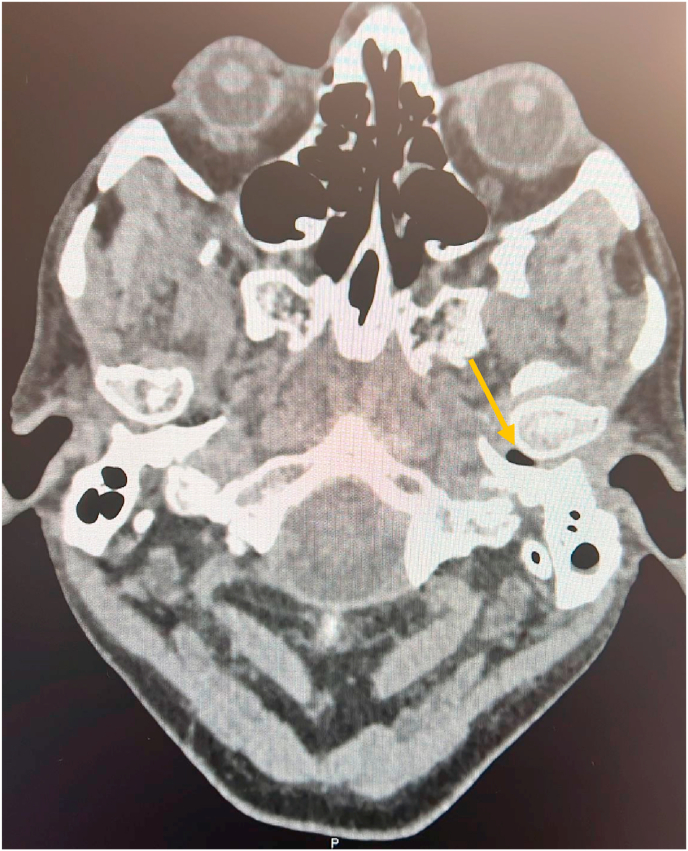
Computerised tomography of the petrous bone. Yellow arrows shows gas bubble posterior to the TMJ on the left side. (For interpretation of the references to colour in this figure legend, the reader is referred to the Web version of this article.)

On examination of the left ear canal, a gas bubble was visible in the distal portion of the EAC which deflated when speaking, producing the audible squeaky sound. There was a small mass just anterior to the tympanic membrane, however, the tympanic membrane was intact. There was no obvious keratinized growth in the attic of the tympanic membrane. His pure tone audiogram was normal. He had CT of temporal bone (Fig. 1.) which showed a 5 mm lesion in the left EAC near the tympanic membrane which was in contact with the anterior inferior canal wall which had features suggesting tissue erosion. Gas bubble was seen in the posterior part of the TMJ likely in relation to the erosion of the EAC. The joint was intact with no arthritic changes (see Fig. 2).

He has started on conservative medical therapy with regular cleaning and debridement of the keratin debris. If the conservative medical management fails to prevent progression of his disease, he will be offered more definitive management, surgery. The surgery involves bony meatoplasty via a postauricular approach for more adequate debridement, canalplasty, and often split-thickness skin grafting to cover the canal defect.

## 3 Discussion

The European Academy of Otology and Neurotology and Japan Otological Society (EAONO/JOS) defined a cholesteatoma as a mass formed by the keratinising squamous epithelium in the tympanic cavity and/or mastoid and subepithelial connective tissue and by the progressive accumulation of keratin debris with or without a surrounding inflammatory reaction. Cholesteatoma is classified into acquired, congenital, and unclassifiable (cholesteatoma whose origin cannot be accurately determined) [5].

Although there has been much debate about the definition and aetiology of cholesteatoma, it is commonly accepted to consider congenital cholesteatoma as an epidermoid cyst behind an intact tympanic membrane, which arises from congenital remnants of keratinising squamous epithelium in the temporal bone [2,6]. It is presumed to be present at birth, and usually diagnosed in childhood [7]. Acquired cholesteatoma might develop from a retraction pocket of the pars flaccida, pars tensa, or both and from basal cell invasion through the basilar membrane and could be a sequela of the dysfunction of middle ear pressure regulation [5]. According to the literature, its incidence varies from 1 to 7.1 cases per 1000 new otologic patients [8,9]. Whereas, External ear canal cholesteatoma has an incident rate of 0.1% worldwide [12].

External ear canal cholesteatoma develops due to the accumulation of squamous keratinized material in the external ear canal. The expansion of external ear canal cholesteatoma leads to the formation of periostitis and sequestrum with inflammatory stimuli of lysosomal enzymes, collagenase, prostaglandins as well as the swelling pressure [13]. However, external ear canal cholesteatoma occurs in the external ear, its further development can damage the anterior wall and inferior wall of the canal as presented^[14]^. In this case, the external ear canal cholesteatoma moves anteriorly and invades anterior bones in the ear canal including the TMJ. The destruction of the anterior bone in the ear canal is visible in the CT scan image. For patients with a high jugular bulb, the development of external ear canal cholesteatoma can easily lead to the jugular bulb exposure^[14]^. If the external ear canal cholesteatoma invades the middle ear, it will cause the same damages as middle ear cholesteatoma does, such as the destruction of facial nerve canal, labyrinth bone, sigmoid sinus and skull base^[13,14]^.

A detailed otological assessment is required to diagnose cholesteatoma, coupled with a full audiometric evaluation and imaging (MRI or CT scan) [11]. The use of auroscope and microscope has allowed an accurate assessment of the tympanic membrane with magnification of the structure. This has resulted in both the early detection and management of the cholesteatoma, which resulted in improved prognosis for patients [10,11].

Treatment of cholesteatoma is dependent on the severity of the disease process [10,11]. Conservative management primarily involves the arrest of disease progression. That is usually obtained by reducing the underlying inflammatory process and preventing the propagation of the disease [11]. Surgical intervention is considered the mainstay of management, and many different approaches have been tailored depending on the size and site of the cholesteatoma.

## 4 Conclusion

EAC cholesteatomas are a rare entity and insidious in nature. It can present with a myriad of clinical manifestations and should be suspected in all patients with unexplained otological and TMJ related signs and symptoms. Temporal bone CT scan can aid the diagnosis and also differentiate the external ear canal from the middle ear cholesteatoma. The late-stage presentations of the disease are very common.

## Ethical approval

N/A.

Consent taken from the patient.

## Sources of funding

None.

## Author contributions

Dr Fatemeh Salimi study concept and design, or interpretation, writing the paper.

Dr Dema Motter data collection, data analysis.

Dr Zahra Salimi contributed to the paper writing.

## Registration of research studies

1. Name of the registry:

2. Unique Identifying number or registration ID:

3. Hyperlink to your specific registration (must be publicly accessible and will be checked):

## Consent

Has been obtained, verbally at the initiation of the case report as well as a signed consent form.

## Guarantor

Fatemeh Salimi.

## Consent for publication

Written informed consent was obtained from the patient's parents for publication of this case report and accompanying images. A copy of the written consent is available for review by the Editor-in-Chief of this journal.

## Provenance and peer review

Not commissioned, externally peer reviewed.

## Declaration of competing interest

None.
